# Growth and Competition of the Tropical Invader *Chromolaena odorata* Are Inhibited by Late Arrival and Mitigated by Nitrogen Addition

**DOI:** 10.3390/plants15182777

**Published:** 2026-09-10

**Authors:** Chunqiang Wei, Saichun Tang, Yumei Pan, Xiangqin Li, Longwu Zhou, Liquan Wei

**Affiliations:** 1 Guangxi Key Laboratory of Plant Conservation and Restoration Ecology in Karst Terrain, Guangxi Institute of Botany, Guangxi Zhuang Autonomous Region and Chinese Academy of Sciences, Guilin 541006, China; wcq@gxib.cn (C.W.); panyumei@gxib.cn (Y.P.); dax5455@163.com (X.L.); zlw@gxib.cn (L.Z.); 2 Guangxi Forest Inventory & Planning Institute, Nanning 530011, China; liquanbeer@163.com

**Keywords:** priority effects, nitrogen addition, growth, competition, relative density, *Chromolaena odorata*

## Abstract

Aims: Understanding how arrival order, nitrogen (N) availability and population density interact to shape plant invasion is critical for predicting and ameliorating biological invasions under global change. *Chromolaena odorata* is one of the most destructive invasive plants in tropical and subtropical regions, but the multifactorial drivers of its establishment and competitive dominance are not fully resolved. Methods: We constructed artificial communities in a common garden experiment and manipulated the arrival order of *C. odorata* (early, simultaneous and late arrival relative to native species), species density (high, medium and low), and N addition (0 vs. 10 g N m^−2^ yr^−1^), and then examined their effects on growth performance and competitive dominance. Results: Early arrival did not increase growth performance of *C. odorata*, but it substantially enhanced the competitive dominance of the invasive species in most communities. However, late arrival caused substantial reductions in height, biomass, and RDI of *C. odorata* across all communities, with the most severe declines observed in treatment groups that did not receive N addition. N addition disproportionately promoted the growth of *C. odorata* regardless of arrival order and enhanced its competitive advantage when it arrived later than native species. In low-density *C. odorata* communities, N addition exhibited the strongest compensatory effect against growth suppression of the invasive species resulting from late arrival. Importantly, when *C. odorata* arrived later than native species in plots receiving N addition, its height and biomass were comparable to plants that arrived at the same time as native species in groups without N addition across most communities. Conclusions: Arrival order is a critical determinant of *C. odorata* invasion success, with early arrival conferring competitive advantages and late arrival imposing severe competitive penalties due to competition with established native species. However, N addition can compensate for late-arrival disadvantages, particularly for low-density *C. odorata*, suggesting that N-enriched ecosystems may remain vulnerable to invasion, even when native communities are established. These results underscore the context-dependent nature of priority effects and highlight that managing colonization timing and nutrient inputs may be effective in similar localized settings, though future field-based, multi-season validation is clearly needed.

## 1. Introduction

Competition from invasive plant species can reduce the diversity of native plant species and alter ecosystem structure and function [1,2]. Resource availability strongly influences both the performance of invasive species and ecosystem processes [3,4]. Global environmental changes, in particular widespread nutrient enrichment, continue to accelerate plant invasion and threaten biodiversity [4,5]. The success of invasive plants is governed by a complex interplay among species traits, environmental conditions, and biotic interactions [6,7,8], yet the temporal dynamics of colonization in communities affected by invasion remain poorly understood.

In natural communities, the order in which invasive species arrive can have a strong impact on community assembly and invasion outcomes through priority effects—the advantages gained by early colonizers that enable them to preempt resources, modify habitat conditions, and suppress colonization by subsequent species [9,10,11]. For invasive species, arriving earlier than native competitors may confer substantial advantages that facilitate their establishment and spread, whereas delayed arrival may limit their success [11,12,13]. These priority effects have increasingly become the focus of investigation as a practical tool that can help control invasions and restore invasion-resistant native plant communities [14,15,16]. However, the role of priority effects in facilitating or resisting plant invasion remains poorly quantified, particularly for aggressive tropical invaders [16]. From this perspective, priority effects can be viewed as transient modulators of niche–fitness ratios, either reinforcing native resistance or facilitating invader dominance depending on trait asymmetries [17]. In addition, the degree to which priority effects influence invasion outcomes is context-dependent [18,19]. The effects of arrival time sometimes vary with native and invasive species density and resource availability [4,20,21], yet the interactive effects between priority effects and local conditions remain largely unexplored.

In addition to its influence on temporal dynamics, resource availability also plays a pivotal role in regulating plant competition and invasion success [3,22]. The fluctuating resource hypothesis (FRH) posits that increased resource availability opens windows for invasion by reducing the competitive resistance of native communities [23]. Anthropogenic nitrogen (N) deposition represents a particularly pervasive form of resource fluctuation [24,25]. N deposition can increase soil nutrient availability, reduce competitive resistance in native communities, and disproportionately advantage fast-growing invasive species with high nutrient acquisition rates [26,27,28]. Resource competition theory, exemplified by the R* rule [29], predicts that elevated N lowers the resource threshold for fast--growing invaders, thereby altering differences in effective fitness between natives and invaders [23,30]. However, a critical unresolved question is whether N addition can compensate for the competitive disadvantages imposed by late arrival. If priority effects and resource availability interact to reinforce one another, ecosystems with abundant N may remain vulnerable to invasion even when native communities are established, because elevated N could enhance the growth and competitive response of late-arriving invaders sufficiently to overcome the early-arrival advantages of native species.

Species density represents another critical factor that modulates invasion outcomes [20,21,31]. Density-dependent processes regulate the intensity of both intra- and interspecific competition, thereby influencing per-capita resource availability, individual growth rates, and population establishment success [31,32,33]. For invasive species, high initial density may increase competitive pressure on resident native species through rapid resource preemption, yet it may also intensify intraspecific competition and reduce per-capita performance [31,34,35]. Conversely, invasive species introduced at low densities may be overwhelmed by native competitors [21,36]. Density dependence interacts with assembly rules by altering the strength of per-capita interactions, which can simultaneously amplify stabilizing and equalizing mechanisms (e.g., through intensified intra- vs. interspecific competition and resource saturation, respectively) [37,38]; the final effect on coexistence, however, depends on the trait asymmetries between native and invasive species [39]. However, it is unclear whether high-density, early-arriving invaders benefit from stronger priority effects than low-density, late-arriving invaders, which may suffer from greater disadvantages when competing with native species. Despite the widely recognized importance of density dependence in population ecology, experimental studies that test the collective effects of arrival order, density, and resource availability remain scarce, limiting our ability to predict invasion outcomes under realistic multifactorial scenarios.

*Chromolaena odorata* (L.) R.M. King & H. Robinson (Asteraceae), native to North, Central, and South America, is one of the most destructive invasive plant species in tropical and subtropical regions, where it can form dense monocultures that suppress native vegetation, alter soil N transformation, and outcompete resident flora [28,40,41]. Its rapid growth, prolific seed production, and allelopathic potential contribute to its competitive superiority [42,43,44]. However, the competitive ability of this invader is context-dependent. For example, fluctuating resource availability can support invasion in communities with low species richness [3] and high light intensity can enhance its competitive advantage over other co-occurring species [45], whereas high phosphorus availability and low light intensity can diminish its competitive advantage [46]. At present, the mechanisms underlying its establishment success in native communities with variable colonization histories remain elusive. Understanding how the timing of *C. odorata* arrival relative to native species with different densities and across a range of nutrient conditions influences its growth and competitive dominance is essential for predicting invasion risk and developing targeted management interventions.

In this study, we conducted a full factorial manipulative experiment that tested the effects of three arrival orders (early, simultaneous and late arrival relative to native species), three relative densities of *C. odorata* (density ratios of *C. odorata* to native species were 29:7, 15:21 and 8:28, designed as high-, medium- and low-density *C. odorata* communities, respectively), and two N addition levels (0 vs. 10 g N m^−2^ yr^−1^). We analyzed how arrival order, N addition, species density, and their interactions affected the growth performance and competitive dominance of *C. odorata* across these communities. We hypothesized that: (i) early arrival would consistently confer growth and competitive advantages to *C. odorata*, while late arrival would suppress its growth and competitive dominance, regardless of species density or N availability; (ii) N addition would increase the growth and competitive dominance of *C. odorata*, regardless of arrival order, and that the magnitude of these effects would differ depending on the arrival order of the invader; this positive effect would be most pronounced for late arrivals, such that the performance of invaders arriving later than native species under N addition would be comparable to that experienced by invaders arriving simultaneously with native species in treatment groups without N addition; and (iii) high-density, early-arriving *C. odorata* would experience stronger priority effects, whereas low-density, late-arriving *C. odorata* would experience greater disadvantage when competing with native species under N addition.

## 2. Results

### 2.1. Growth Performance of C. odorata

#### 2.1.1. Plant Height and Total Biomass

Arrival order, species density, and N addition significantly affected *C. odorata* plant height and total biomass. Additionally, the interaction between arrival order and density and between density and N addition significantly influenced total biomass (Table 1).

**Figure 1:**
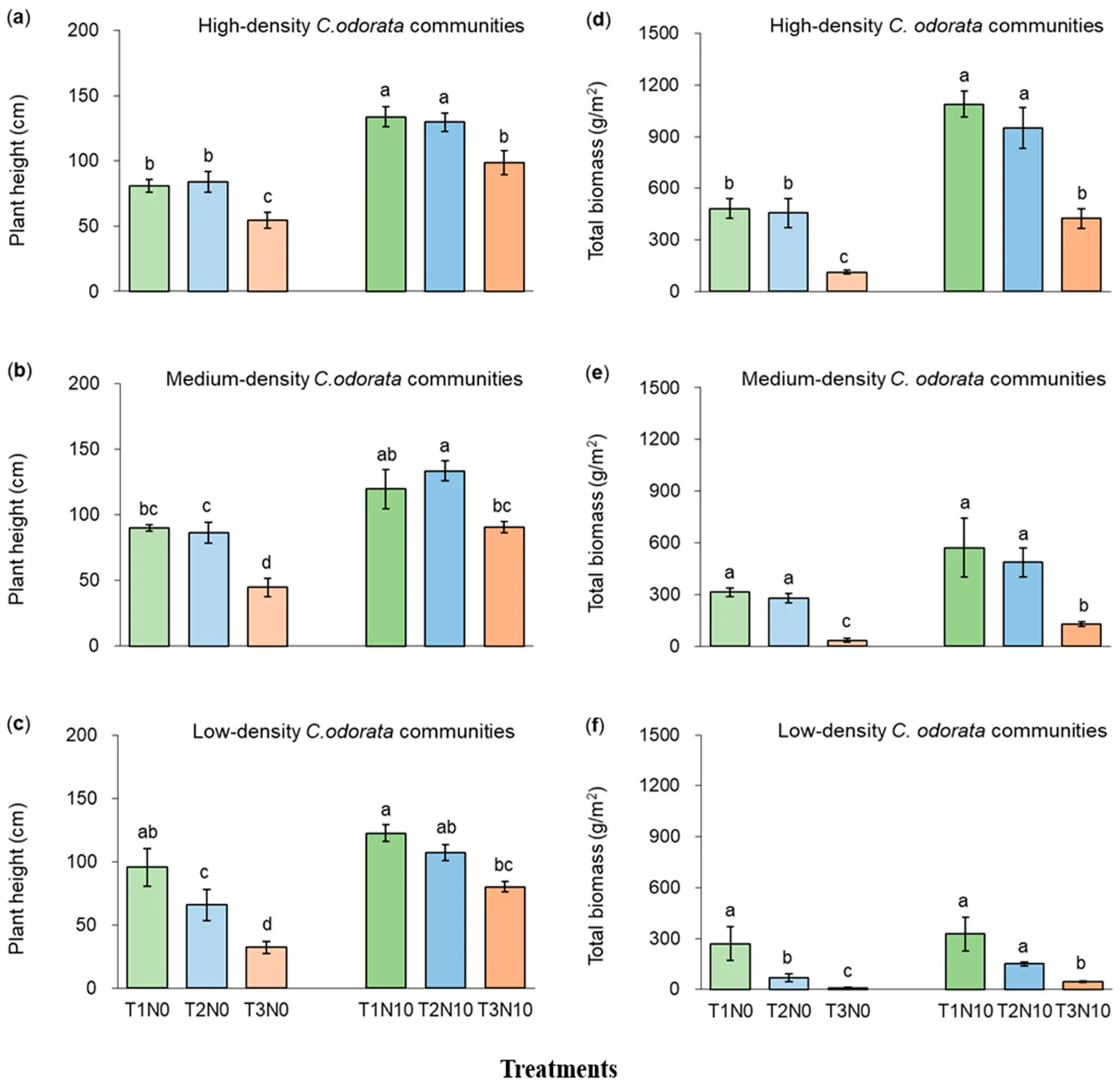
*C. odorata* plant height and total biomass in high-, medium-, and low-density communities (density ratios of *C. odorata* to native species were 29:7, 15:21 and 8:28, respectively) under different arrival order scenarios (T1N0: early arrival without N addition; T2N0: simultaneous arrival without N addition; T3N0: late arrival without N addition; T1N10: early arrival with N addition; T2N10: simultaneous arrival with N addition; T3N10: late arrival with N addition). Values are means ± SE of the raw data. Different lowercase letters above bars indicate significant differences among treatments (*p* < 0.05), as determined by ANOVA on log-transformed (**b**,**e**,**f**), square root-transformed (**c**), and untransformed (**a**,**d**) data, respectively.

When *C. odorata* arrived earlier than native species (T1), its height did not differ significantly from that in T2 (simultaneous arrival) in any community (Figure 1a–c) except in low-density *C. odorata* communities without N addition (N0), where plants were 45% taller than in T2 (Figure 1c). However, when *C. odorata* arrived later than native species (T3), plant height decreased significantly across all communities (Figure 1a–c), except in low-density *C. odorata* communities under N addition (N10). Without N addition, the reductions were 35%, 48%, and 51% (T3N0 vs. T2N0) in high-, medium-, and low-density *C. odorata* communities, respectively. Under N addition (N10), the corresponding decreases were 24% and 32% in high- and medium-density communities (T3N10 vs. T2N10), respectively.

The addition of N significantly increased *C. odorata* plant height in high-density communities across all arrival order treatments, with increases of 66%, 55%, and 81% under T1, T2, and T3, respectively (T1N0 vs. T1N10, T2N0 vs. T2N10, and T3N0 vs. T3N10; Figure 1a). Significant increases were also observed in medium-density communities (+55% in T2 and +103% in T3; Figure 1b) and low-density communities (+63% in T2 and +148% in T3; Figure 1c). Across all communities, the greatest increase in plant height was recorded under T3. There were no significant differences in plant height between T3N10 and T2N0 across all communities (Figure 1a–c). In general, N addition mitigated the disadvantage in plant height caused by late arrival.

Total biomass in the T1 group was not significantly different from that of T2 in any community (Figure 1d–f), except in low-density communities without N addition, where total biomass was 291% greater than in T2 (Figure 1f). However, total biomass under T3 decreased significantly across all communities compared with that in T2 (Figure 1d–f). Without N addition, the reductions were 75%, 88%, and 86% (T3N0 vs. T2N0) in high-, medium-, and low-density communities, respectively; with N addition, the corresponding decreases were 55%, 74%, and 69% (T3N10 vs. T2N10), respectively.

The addition of N substantially increased the total biomass of *C. odorata* in high-density communities across all arrival order treatments, by 126%, 108%, and 274% under T1, T2, and T3, respectively (T1N0 vs. T1N10, T2N0 vs. T2N10, and T3N0 vs. T3N10; Figure 1d). Total biomass also increased (+280%) under N addition in medium-density communities in the T3 group (Figure 1e), and by 117% and 397% in low-density communities under T2 and T3, respectively (Figure 1f). Moreover, total biomass did not differ significantly between T3N10 and T2N0 in high- and low-density communities (Figure 1d,f). Overall, N addition mitigated the negative effect of late arrival on total biomass.

#### 2.1.2. SPAD Value and Leaf N Content

**Figure 2:**
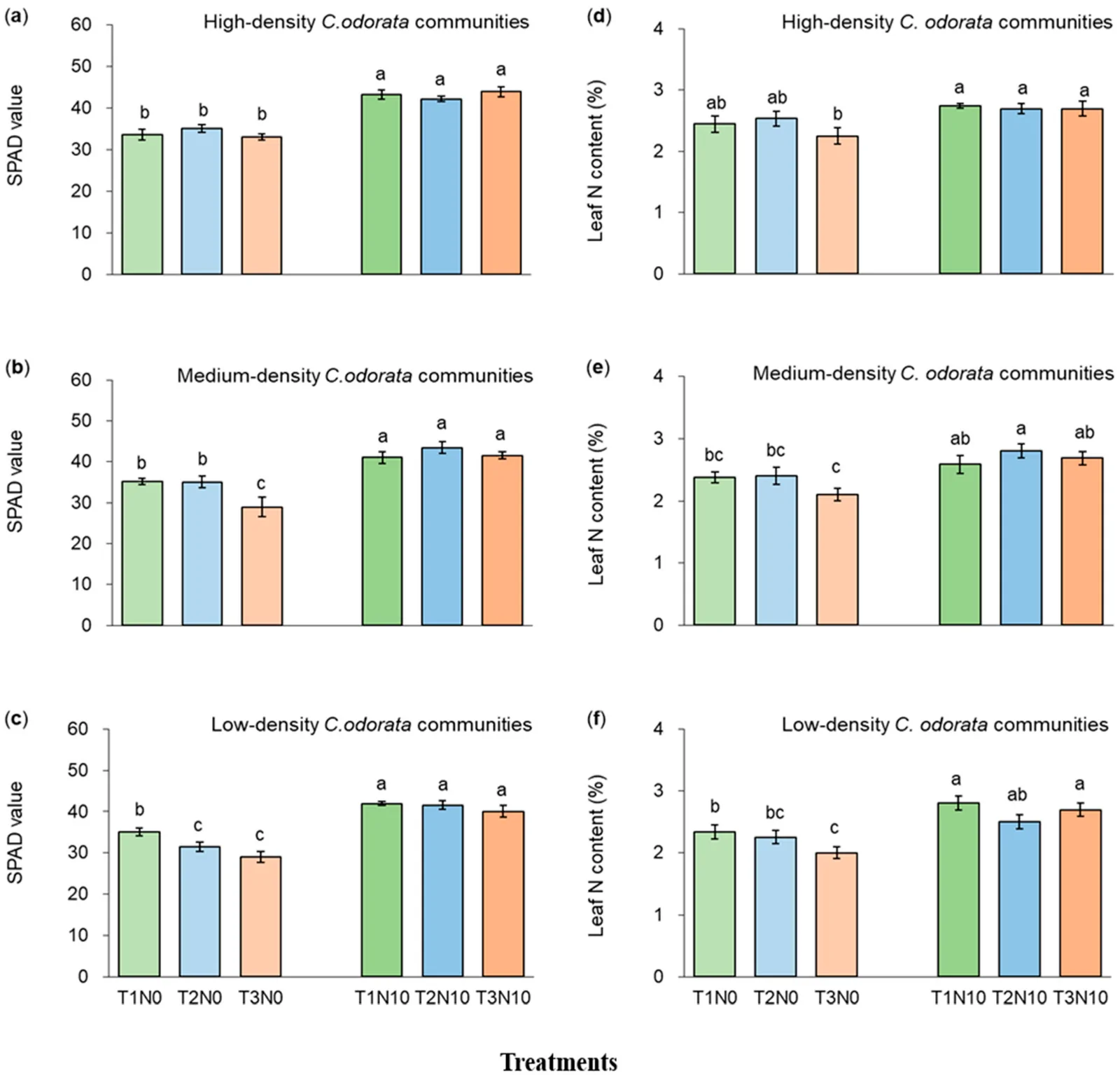
*C. odorata* SPAD values and leaf N content in high-, medium-, and low-density communities (density ratios of *C. odorata* to native species were 29:7, 15:21 and 8:28, respectively) under different arrival order scenarios (T1N0: early arrival without N addition; T2N0: simultaneous arrival without N addition; T3N0: late arrival without N addition; T1N10: early arrival with N addition; T2N10: simultaneous arrival with N addition; T3N10: late arrival with N addition). Values are means ± SE of the raw data. Different lowercase letters above bars indicate significant differences among treatments (*p* < 0.05).

Arrival order, density, and N addition significantly affected *C. odorata* SPAD; N addition also significantly affected leaf N content (Table 1). The addition of N also significantly increased SPAD across all density levels and arrival order treatments (Figure 2a–c). *C. odorata* SPAD was lowest in T3N0 across all communities, significantly lower than in the N-addition communities (Figure 2a–c). Similarly, leaf N content was lowest in T3N0, significantly lower than in communities with N addition (Figure 2d–f).

### 2.2. Competitive Dominance of C. odorata

Arrival order, density, and their interaction, as well as the interactions between density and N addition and between arrival order and N addition, all significantly affected *C. odorata* RDI (Table 1).

**Figure 3:**
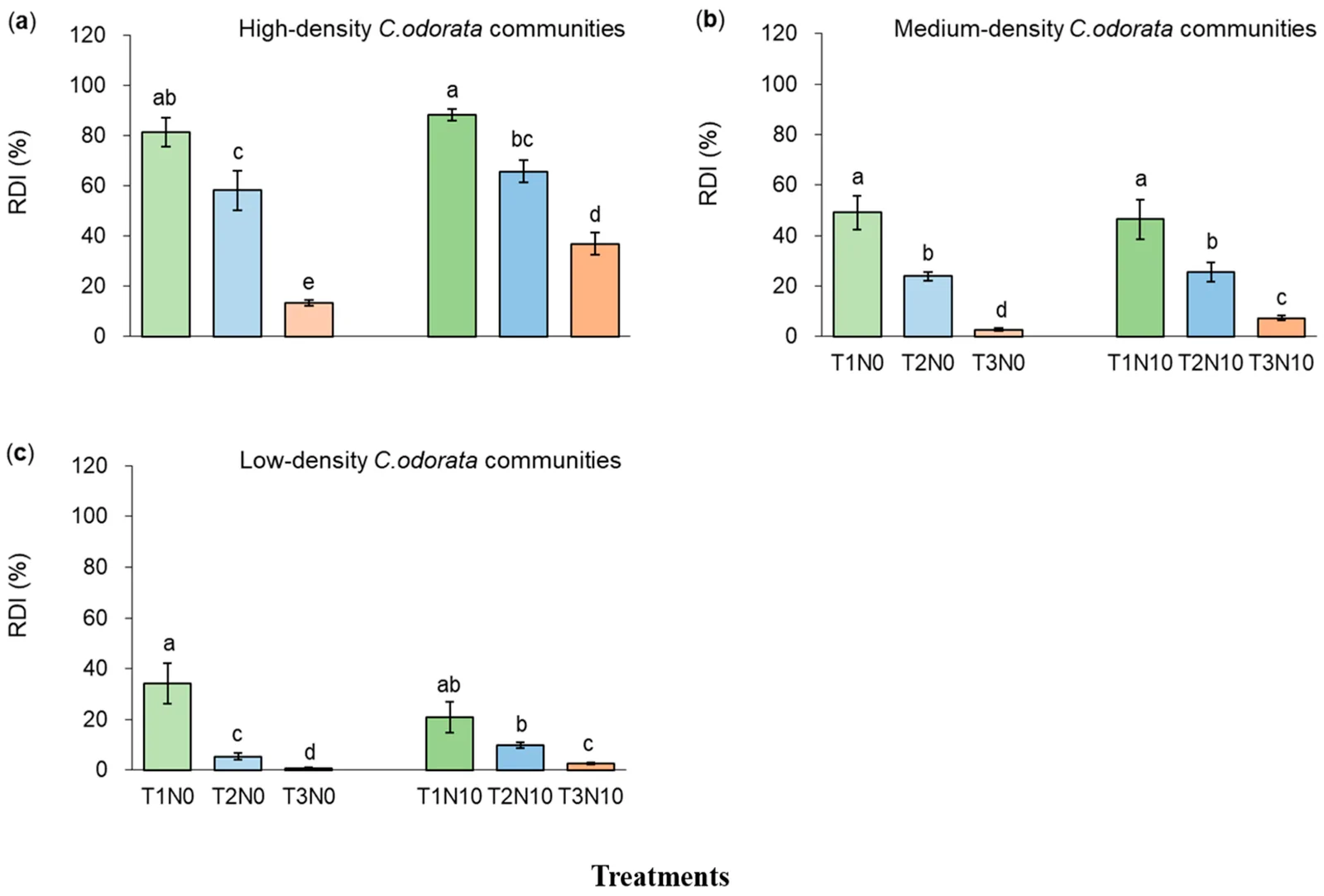
Relative dominance index (RDI) of *C. odorata* in high-, medium-, and low-density communities (density ratios of *C. odorata* to native species were 29:7, 15:21 and 8:28, respectively) under different arrival order scenarios (T1N0: early arrival without N addition; T2N0: simultaneous arrival without N addition; T3N0: late arrival without N addition; T1N10: early arrival with N addition; T2N10: simultaneous arrival with N addition; T3N10: late arrival with N addition). Values are means ± SE of the raw data. Different lowercase letters above bars indicate significant differences among treatments (*p* < 0.05), as determined by ANOVA of log-transformed data (**a**–**c**).

Values of RDI in the T1 group increased significantly across all communities compared with T2 (Figure 3a–c), except under T1N10 in low-density communities (Figure 3c). Without N addition, increases in RDI in high-, medium-, and low-density communities were 40%, 106%, and 527%, respectively (T1N0 vs. T2N0); under N addition, RDI increased only in high- and medium-density communities, by 34% and 81%, respectively (T1N10 vs. T2N10). Conversely, RDI decreased significantly in all communities under T3 compared to T2. Without N addition, RDI fell by 77%, 89%, and 86% in high-, medium-, and low-density communities, respectively (T3N0 vs. T2N0); with N addition, the corresponding reductions were 44%, 71%, and 74% (T3N10 vs. T2N10).

We did not observe increases in RDI under N addition in T1 (T1N0 vs. T1N10) or T2 (T2N0 vs. T2N10) except in low-density communities under T2 (Figure 3a–c). However, N addition substantially increased RDI in T3 across all communities (T3N0 vs. T3N10), increasing by 177%, 173%, and 228% in high-, medium-, and low-density communities, respectively (Figure 3a–c). N addition partly compensated for the competitive disadvantage caused by late arrival.

**Figure 4:**
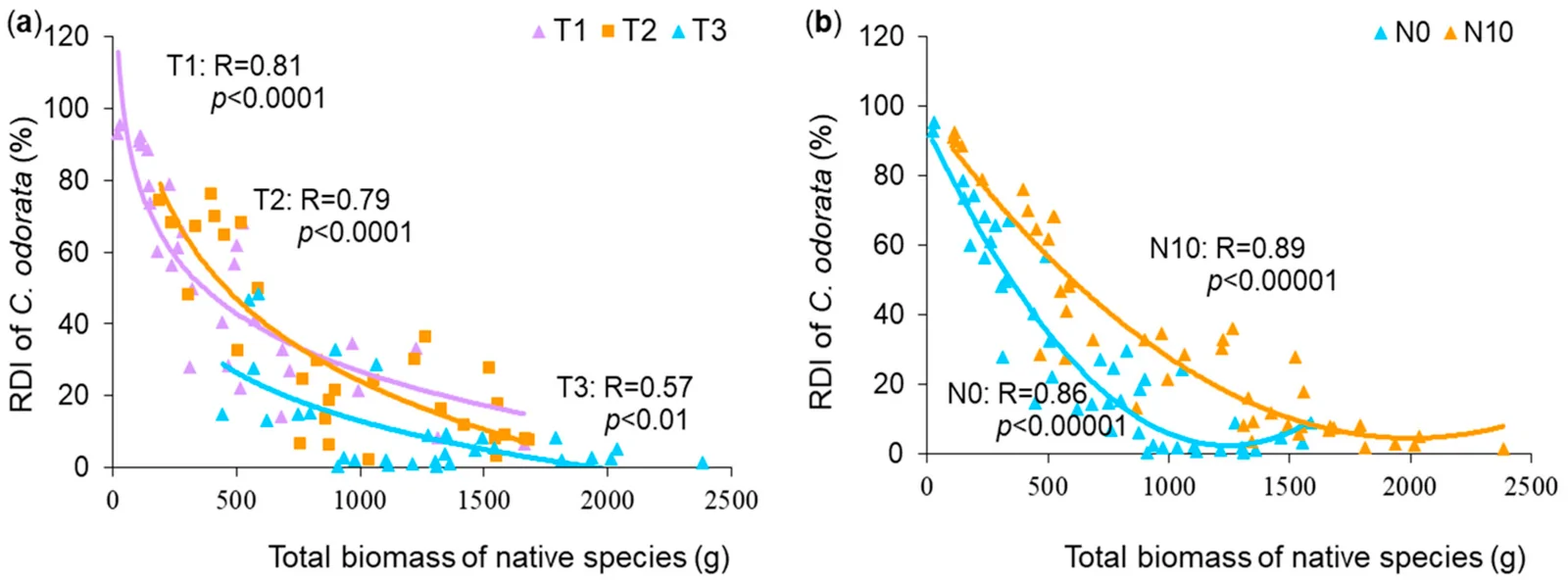
Relationship between *C. odorata* relative dominance index (RDI) values and the total biomass of native species under different arrival order scenarios (**a**) and under different N addition treatments (**b**).

The RDI of *C. odorata* was significantly and negatively related to native species biomass (Figure 4a,b). For a given native plant biomass, the RDI of *C. odorata* was higher in T1 and T2 than in T3 (Figure 4a); moreover, it was higher in communities with N addition than in those without (Figure 4b). In addition, the total biomass of native species was significantly lower in T1 than in T2 and T3 (Figure S1).

## 3. Discussion

We investigated how arrival order, density, N addition, and their interactions affected the growth and competitive dominance of the tropical invasive species *C. odorata* in constructed communities in a common garden experiment. We found that arrival order was a critical determinant of *C. odorata* growth performance and competitive dominance in native communities. Furthermore, we found that the magnitude of priority effects was context-dependent, varying among *C. odorata* density treatments under different N application levels.

### 3.1. Effects of Arrival Order on Growth and Competitive Dominance of C. odorata

In our experiment, early arrival of *C. odorata* generally did not result in greater plant height or biomass production compared with simultaneous arrival, except in low-density communities without N addition. In high- and medium-density communities, *C. odorata* experienced relatively weak competitive suppression from native plants due to its own high relative abundance. Consequently, early arrival conferred little additional benefit because competitive pressure was already low. By contrast, in low-density communities without N addition, *C. odorata* suffered severe competitive suppression from the high-density native plants. Under these conditions, early arrival weakened competitive suppression and thereby significantly enhanced *C. odorata* growth. These results suggest that priority effects on invader growth are context-dependent, with the most pronounced effects occurring when the invader experiences an initial competitive disadvantage. However, early arrival significantly increased the RDI of *C. odorata* across all communities (by 34–527%). This increase likely resulted from reduced total biomass of native species, which was significantly lower when *C. odorata* arrived early than when it arrived simultaneously. This suggests that early-arriving *C. odorata* gained a competitive advantage by preempting resources and suppressing the growth of later-arriving native species, a finding consistent with previous studies showing that early arrival promotes the competitive dominance of invasive species [12,47,48]. However, this result contradicts the findings of Matos and Ward (2025) [49], who reported that invasive grass did not exhibit priority effects. This outcome partly supports our first hypothesis. The substantial increase in RDI under early arrival, particularly in low-density *C. odorata* communities (density ratio of *C. odorata* to native species = 8:28) without N addition (+527%), provides additional evidence that early arrival can enhance the competitive dominance of invasive species when competition for resources is intense.

Consistent with our hypothesis, late arrival negatively affected the growth performance and competitive dominance of *C. odorata* across all densities and N addition treatments. When the invasive species arrived after the native species, its height declined by 24–51%, total biomass by 55–88%, and RDI by 44–89% in high-, medium-, and low-density *C. odorata* communities. This suggests that late-arriving *C. odorata* suffered competitive inhibition from established native species, a finding similar to those of previous studies showing that arrival order affects the success of invasive species such as *Bromus tectorum*, *Ambrosia trifida*, and *Senecio inaequidens* [10,11,12]. These results are also consistent with a recent meta-analysis demonstrating that invasive species benefit from arriving early and that delayed invasion can significantly affect their performance [13]. At the time of harvest in October, individuals of *C. odorata* in all three treatments (early-arrival, simultaneous-arrival, and late-arrival) were in the late vegetative growth phase, and their growth had markedly slowed. Consequently, the additional 21 days of growth in the early-arrival and simultaneous-arrival treatments, compared with the late-arrival treatment, likely contributed only minimally to their final biomass. Thus, although growth duration had a limited effect on the final performance of *C. odorata*, arrival order emerged as a critical determinant of its invasion success.

Niche preemption and modification are the two main categories of mechanisms underlying priority effects [9]. Niche preemption occurs when early-arriving species inhibit later arrivals by reducing the availability of resources such as nutrients, space, and light [9]; niche modification occurs when early arriving species alter the types of niches available to later arrivals, thereby affecting which species can establish populations [9]. For example, soil fertilization by leguminous species may facilitate later arrivals, whereas the exudation of allelochemicals may inhibit them by altering their growth rates [9,50]. In our experiment, the relative importance of these two mechanisms remains to be clarified. Previous studies have reported that *C. odorata* exhibits allelopathic effects on native Chinese species [42,43]. However, seedlings of the six native species used in our experiment were tolerant to the allelopathic effect of *C. odorata* leaf aqueous extract [51]. This suggests that a niche modification effect driven by *C. odorata* allelopathy is unlikely to account for the observed patterns. Instead, our results provide multiple lines of evidence pointing toward niche preemption. The near-complete competitive exclusion of late-arriving species, coupled with their reduced nitrogen (N) content, aligns with the resource-depletion signature characteristic of preemption [9,12]. In our results, the significant negative correlation between *C. odorata* RDI and native total biomass suggests that as native species accumulated greater biomass (i.e., occupied more space and drew down more resources), the competitive performance of the late-arriving *C. odorata* was proportionally suppressed. Furthermore, in the treatment without N addition, *C. odorata* that arrived later exhibited lower SPAD values and leaf N content. These physiological declines directly reflect reduced soil N availability caused by prior native uptake, which is a hallmark of resource preemption rather than chemical modification of the niche. Nevertheless, while these indicators implicate niche preemption as the dominant driver of priority effects in our system, we cannot definitively exclude the possibility that native species exerted allelopathic effects on *C. odorata*, as we did not test this direction of interaction. Future studies are needed to fully disentangle the relative contributions of resource competition and biochemical interference.

### 3.2. Effects of N Addition on the Growth and Competitive Dominance of C. odorata

*Chromolaena odorata* can alter soil N transformations and has high N utilization ability [28,40]. Our results were largely consistent with our second hypothesis. N addition disproportionately stimulated the growth of *C. odorata* regardless of arrival order. Moreover, this effect of N significantly strengthened the invader’s competitive ability when it arrived later than the native species. These results are in accordance with previous studies reporting that increased N deposition benefits fast-growing invasive species [4,25,27]. Pan et al. (2024) [52] also reported that high N addition promoted the growth and competitive ability of *C. odorata* in a karst region.

Under N addition, the greatest increases in the growth and competitive dominance of *C. odorata* plants were observed when it arrived after the native species. Under these conditions, plant height increased by 81–148%, total biomass by 274–397%, and RDI by 173–228% across high-, medium-, and low-density *C. odorata* communities. We also found that reductions in growth and competitive dominance caused by late arrival were substantially attenuated under N addition. Moreover, plant height and total biomass of late-arriving *C. odorata* under N addition were comparable to those when *C. odorata* arrived at the same time as native species but without N addition. These results are consistent with the interpretation that higher N availability may alleviate resource limitation and thereby enhance the competitive ability of the invasive species [53], even when it arrives after the native species. However, we must emphasize that this interpretation remains speculative in the context of our study, because we did not directly measure soil nitrogen pools, nitrogen transformation rates, or plant physiological traits related to nitrogen uptake and utilization. If this proposed mechanism is validated, it would suggest that N addition may mitigate some of the disadvantages associated with late arrival. This is consistent with work by Xu et al. (2025) [4], which found that N enrichment reduced phenological niche differences between the late-growing exotic plant *Spartina alterniflora* and resident native species, thereby facilitating invasion. Invasive species can invade resident communities due to their strong competitive ability and rapid growth [54]. Our results suggest that N deposition in natural ecosystems may facilitate *C. odorata* invasion, potentially by alleviating competitive disadvantages associated with late arrival, although the underlying mechanisms require further investigation. Thus, in addition to prioritizing early arrival of native species, reducing N availability may be a strategy that can help control *C. odorata* invasion and restore invasion-resistant native plant communities, provided that the proposed nitrogen-driven mechanism is confirmed by targeted measurements in future studies.

It should be noted that the timing of N addition initiation differed among treatments: the early-arrival and simultaneous-arrival treatments had a 40-day N-free establishment period, whereas the late-arrival treatment received N after only 19 days. This asynchrony implies that plants in the different treatments were likely at different developmental stages when N addition commenced. However, Zheng et al. (2020) [3] found that, in communities with high native species diversity, nutrient fluctuation—which inherently creates intermittent low-nutrient periods—did not significantly affect the invasion success of *C. odorata*. This suggests that *C. odorata* may be relatively insensitive to such temporal differences in nutrient supply, although we cannot completely rule out its influence. We therefore acknowledge this limitation and advise caution when interpreting comparisons involving the late-arrival treatment.

### 3.3. Effects of Density on the Growth and Competitive Dominance of C. odorata

The number of invading individuals at a single location represents one aspect of propagule pressure [55]. High densities of invasive species may increase competitive pressure on native residents [34]. Byun et al. (2015) [36] showed that a greater density of resident native species can reduce the success of invasion by *Phragmites australis*, especially when the propagule pressure of the invader was low. Zheng et al. (2017) [56] also found that high-density native plants from the origin of *C. odorata* can suppress the growth of this invasive species.

In our experiment, we found that *C. odorata* RDI values increased with increasing density. However, low-density *C. odorata* showed the strongest response to early arrival. Early arrival did not increase the growth performance of *C. odorata* in high- and medium-density *C. odorata* communities. In contrast, early arrival enhanced the plant height (+45%), biomass (+291%) and RDI (+527%) in low-density *C. odorata* communities without N addition compared to simultaneous arrival treatment groups. This is contrary to our third hypothesis. This density-dependent pattern suggests that the benefits of priority effects are not uniformly distributed but are critically modulated by intraspecific crowding [17]. The greater benefit of early arrival for low-density *C. odorata* can be explained by a self-dilution effect resulting from intraspecific competition. High density creates intense internal crowding among early arrivers [17,30] and thus diminishes priority advantage at the individual level. By contrast, at low densities, reduced intraspecific competition enables early colonists to monopolize resources and capitalize on the window of opportunity prior to native establishment [30,57].

We also found that low-density *C. odorata* gained greater benefits from N addition than high-density *C. odorata* under late arrival. The addition of N produced the strongest compensatory effect against the growth suppression caused by late arrival in low-density *C. odorata* communities (148%, 397%, and 228% increases in plant height, biomass, and RDI, respectively). In low-density *C. odorata* communities, the competition faced by the plants is mainly interspecific. Compared with native species, *C. odorata* generally has higher resource use efficiency and growth rate [58], and N addition further enhances its growth and competitive ability. When *C. odorata* arrived late, N addition replenished soil resources depleted by earlier-established natives, weakening niche preemption. The pronounced height gain allowed late-arriving individuals to reduce light limitation, which subsequently drove substantial biomass accumulation and promoted dominance of the invader in the community. Hence, under low-density conditions, N addition reduced the disadvantage of late arrival. In high-density *C. odorata* communities, conspecific individuals have completely overlapping niches (i.e., identical requirements for N, light, and water), so the plants face strong intraspecific competition. Although N addition increases total resources, these resources are diluted among many conspecific individuals, and mutual inhibition among individuals reduces the positive effect of N addition. These results suggest that in N-rich environments, even sparse, late-arriving propagules can pose a substantial invasion risk, as nutrient pulses can amplify individual performance beyond competitive expectations.

Recent work suggests that sowing diverse native species at high seed densities, together with the priority effects associated with early arrival, can result in the competitive exclusion of the common invasive species *Ageratina altissima* [20]. Therefore, giving native species priority and increasing the density and diversity of native species may help control invasion by *C. odorata* and support the restoration of native communities.

We acknowledge that our garden-based experimental setting and short duration impose limitations on ecological interpretation, as the controlled environment lacked natural soil heterogeneity, biotic interactions (e.g., herbivores, pathogens), and climatic variability, which may alter competitive dynamics in natural communities. Therefore, while our results indicate that N addition enhanced *C. odorata* competitive performance and attenuated late-arrival disadvantages under these simplified conditions, these findings should be interpreted as indicative of potential competitive mechanisms rather than as direct evidence that N deposition increases field invasion risk. Natural invasion success depends on additional biotic and abiotic factors—including seed dispersal, herbivory, soil heterogeneity, and long-term community dynamics—that were not captured here. Long-term field experiments are needed to confirm whether these competitive outcomes persist in more complex, realistic ecosystems. If future studies corroborate these patterns, reducing N inputs alongside native species restoration may offer a complementary approach for managing *C. odorata* invasion.

## 4. Materials and Methods

### 4.1. Site Description and Plant Materials

This experiment was conducted in a common garden in Guangxi Institute of Botany, Guilin City, Guangxi Zhuang Autonomous Region, China. The study region has a subtropical monsoon climate, with mean annual rainfall of 1900 mm. The mean annual temperature is 19 °C, with the coldest month (January) averaging 8 °C and the warmest month (July) averaging 28 °C.

*Chromolaena odorata* is designated as a first-class invasive plant species in China [59]. Since its introduction to Guangxi in the early 1990s, it has spread rapidly and colonized diverse habitats, often forming dense monocultures or mixed communities with native grass-shrub complexes. These invasions displace native species and damage vegetation in natural ecosystems in Guangxi. In this study, we examined how the arrival order of *C. odorata* relative to native species (early, simultaneous, or late) affects its growth and competitive ability across varying conspecific densities under N addition. We established artificial communities in the common garden in April 2024 using seven native species from *C. odorata*-invaded habitats. The seven species were *Anisomeles indica* (L.) Kuntze, *Senna tora* (L.) Roxb., *Neyraudia reynaudiana* (Kunth) Keng ex Hitchc., *Vitex negundo* L., *Bidens biternata* (Lour.) Merr. & Sherff, *Boehmeria nivea* (L.) Gaudich., and *Caryopteris incana* (Thunb.) Miq. From October to December 2023, we collected seeds of *C. odorata* and the seven native species from locations near Guohua Town, Pingguo County, Guangxi Zhuang Autonomous Region, China. Seeds were stored in paper bags at room temperature.

### 4.2. Experimental Design

We constructed artificial communities using *C. odorata* and the seven native species described above. In these communities, we manipulated the arrival order of *C. odorata* (early, simultaneous, or late relative to native species), plant density (high, medium, or low), and N addition (0 vs. 10 g N m^−2^ yr^−1^). Each treatment was replicated five times and represented a unique combination of arrival order, N treatment, and species density, resulting in 90 constructed communities.

#### 4.2.1. Arrival Order Treatments

Following Delory et al. (2019) [12] and Ulrich and Perkins (2014) [60], we used a 21-day arrival interval between *C. odorata* and native species to establish priority treatments. For the early-arrival treatment (T1), we planted *C. odorata* seedlings in the designated communities on 22 May 2024 and allowed them to establish for 21 days. Native seedlings were then transplanted to their corresponding communities on 13 June 2024. For the simultaneous-arrival treatment (T2), seedlings of both native species and *C. odorata* were planted in each community on the same date (22 May 2024). For the late-arrival treatment (T3), we followed the same procedure as T1 but reversed the arrival order.

#### 4.2.2. Density Treatments

According to our earlier soil seed bank germination study, *C. odorata* exhibited an extended germination period from March to August. The relative density of invader seedlings (*C. odorata* seedlings as a proportion of all seedlings) in Guangxi showed strong seasonal variation, with a pronounced peak in May (73%), substantial levels in April and June (30–45%), and lower values in early spring (13%) and late summer (24%). Based on these results, we established three community types with *C. odorata*-to-native species ratios of 29:7, 15:21, and 8:28. These ratios corresponded to relative densities of approximately 81%, 42%, and 22%, respectively, which were designated as high-, medium-, and low-density *C. odorata* communities. Each community contained 36 individuals in a 1 × 1 m plot, with adjacent plots separated by 1 m. In the high-density *C. odorata* communities, we planted 29 *C. odorata* seedlings and 7 native seedlings (one per species); in the medium-density *C. odorata* communities, 15 *C. odorata* seedlings and 21 native seedlings (three per species); and in the low-density *C. odorata* communities, 8 *C. odorata* seedlings and 28 native seedlings (four per species). In total, we established 90 communities. In the first ten days, a few seedlings died and were immediately replaced with seedlings of similar size. All communities received 5 L of water per day for the first 15 days, after which they were maintained under natural conditions. Non-target weeds were removed as needed.

#### 4.2.3. Nitrogen Addition Treatments

Communities were divided into two groups: one received N additions (N10) and the other did not (N0). N addition began on 2 July 2024 after seedlings were established. The N deposition rate is as high as 65 kg ha^−1^ yr^−1^ in the part of South-Central China adjacent to Guangxi [61] and is projected to rise to 105 kg ha^−1^ yr^−1^ in Asia by 2030 [62]. To simulate this future scenario, we added N at a rate of 10 g m^−2^ yr^−1^ (equivalent to 100 kg ha^−1^ yr^−1^) using the procedure described in Liu (2011) [63]. Consistent with widespread approaches to simulating atmospheric N deposition, we supplemented experimental plots with NH_4_NO_3_. Following He et al. (2012) [25], we applied N as NH_4_NO_3_ dissolved in deionized water. A total of 28.6 g of NH4NO3 was divided into three equal portions and added to each N-addition community on 2 July, 23 July, and 12 August 2024, respectively. The non-N-addition groups received an equal volume of water at each corresponding time point.

#### 4.2.4. Data Collection and Analysis

In late August, we selected fully expanded, mature leaves from five randomly selected plants from each treatment to measure relative chlorophyll content (SPAD) using a SPAD-502 chlorophyll meter (Konica Minolta, Inc., Tokyo, Japan). We collected a second set of mature leaves, dried them to constant weight, ground them, and measured leaf N content using the Kjeldahl method. Prior to harvest, we measured plant height. In early October, we harvested all plants, washed their roots with tap water, and separated them into belowground (roots) and aboveground (shoots, leaves, and flowers) portions. These portions were then dried at 70 °C to constant weight and weighed.

We analyzed plant growth performance using plant height, total biomass (the sum of belowground and aboveground biomass), SPAD values, and leaf N content, as well as competitive dominance of *C. odorata*. Following Yuan et al. (2013) [64] and Zhang et al. (2017) [65], we used the relative dominance index (RDI) as a proxy for competitive dominance. We selected RDI as the primary metric because it integrates both plant size and abundance, providing a comprehensive measure of species dominance in mixed communities. This index has been widely applied in invasion ecology to evaluate the competitive success of invasive species in multispecies assemblages [3,64,65]. We calculated these values as RDI = [*C. odorata* biomass/(*C. odorata* biomass + total native species biomass)] × 100%. The RDI ranges from 0% to 100%, with higher values indicating greater competitiveness of *C. odorata* [65].

A three-way analysis of variance (ANOVA) was conducted to examine the main and interactive effects of arrival order, density, and N addition on the growth performance (plant height, total biomass, SPAD, and leaf N content) and the relative dominance index (RDI) of *C. odorata*. The full factorial model comprised all main effects, three two-way interaction terms (arrival order × density, arrival order × N, and density × N), and the three-way interaction term (arrival order × density × N). All factors were treated as fixed effects within the univariate general linear model (GLM) framework. One-way ANOVA and the least significant difference (LSD) post hoc test were used to test for differences in these variables among treatments within each density level. Prior to parametric analyses, we checked the model residuals for normality and homogeneity of variances on the model residuals using the Shapiro–Wilk test and Levene’s test, respectively. If data were non-normally distributed, log-transformed or square-root-transformation was applied to improve the normality and homogeneity of variance. Linear and non-linear regression were used to examine the relationship between *C. odorata* competitive dominance (RDI) and the total biomass of native species, respectively. All analyses were carried out using SPSS version 27.0.

## 5. Conclusions

In our short-term garden experiment with artificially assembled communities over a single growing season, arrival order emerged as a critical determinant of *C. odorata* invasion success. Early arrival conferred competitive advantages, whereas late arrival incurred severe competitive penalties due to interactions with established native species. Within this experimental framework, N addition partially compensated for the disadvantages of late arrival, particularly at low *C. odorata* densities, suggesting that even under constrained conditions, N-enriched systems may remain vulnerable to invasion after native communities are established. However, given that our findings are restricted to controlled garden mesocosms, a single growing season, and synthetic species assemblages, we urge caution in interpreting the broader claim that N enrichment universally increases invasion risk in natural ecosystems. Nevertheless, our results underscore the context-dependent nature of priority effects and highlight that managing colonization timing and nutrient inputs may be effective in similar localized settings, though future field-based, multi-season validation is clearly needed.

## Figures and Tables

**Table 1:** Three-way ANOVA results for the effects of arrival order (T), density (D), N addition (N), and their interactions on plant growth and competitive dominance.

Source	df	Plant Height		Total Biomass		SPAD Value		Leaf N Content		RDI	
		F	Partial η^2^	F	Partial η^2^	F	Partial η^2^	F	Partial η^2^	F	Partial η^2^
T	2	41.49 ***	0.54	45.04 ***	0.56	6.24 **	0.15	2.93	0.08	134.25 ***	0.79
D	2	3.86 *	0.10	58.12 ***	0.62	4.12 *	0.10	1.97	0.05	156.38 ***	0.81
N	1	118.46 ***	0.62	49.60 ***	0.41	257.68 ***	0.78	54.51 ***	0.43	3.26	0.04
T × D	4	1.88	0.09	3.37 *	0.16	2.39	0.12	1.00	0.05	8.60 ***	0.32
T × N	2	0.58	0.02	1.97	0.05	4.57 *	0.11	3.09	0.08	3.14 *	0.08
D × N	2	0.46	0.01	12.93 ***	0.26	0.04	0.00	0.85	0.02	4.52 *	0.11
T × D × N	4	0.57	0.03	0.46	0.03	1.16	0.06	0.50	0.03	0.80	0.04

Levels of significance: * *p* < 0.05, ** *p* < 0.01, *** *p* < 0.001. RDI: relative dominance index; SPAD: relative chlorophyll content.

## Data Availability

The original contributions presented in the study are included in the article; further inquiries can be directed to the corresponding author.

## Supplementary Materials

The following supporting information can be downloaded at: https://www.mdpi.com/article/10.3390/plants15182777/s1. Figure S1. Total biomass of native species in high-, medium-, and low-density *C. odorata* communities (density ratios of *C. odorata* to native species were 29:7, 15:21, and 8:28, respectively) under different arrival order scenarios.

## Abbreviations

The following abbreviations are used in this manuscript:

SPAD	Relative chlorophyll content
RDI	Relative dominance index
