# Supplementary material for: Growth and Competition of the Tropical Invader Chromolaena odorata Are Inhibited by Late Arrival and Mitigated by Nitrogen Addition
Source: Plants (Basel). 2026 Sep 10;15(18):2777. doi: 10.3390/plants15182777 (PMC13610280; doi:10.3390/plants15182777)
Supplement: Supplementary file 1 [file plants-15-02777-s001.zip › plants-4467879-supplementary.pdf]

Supplementary Material

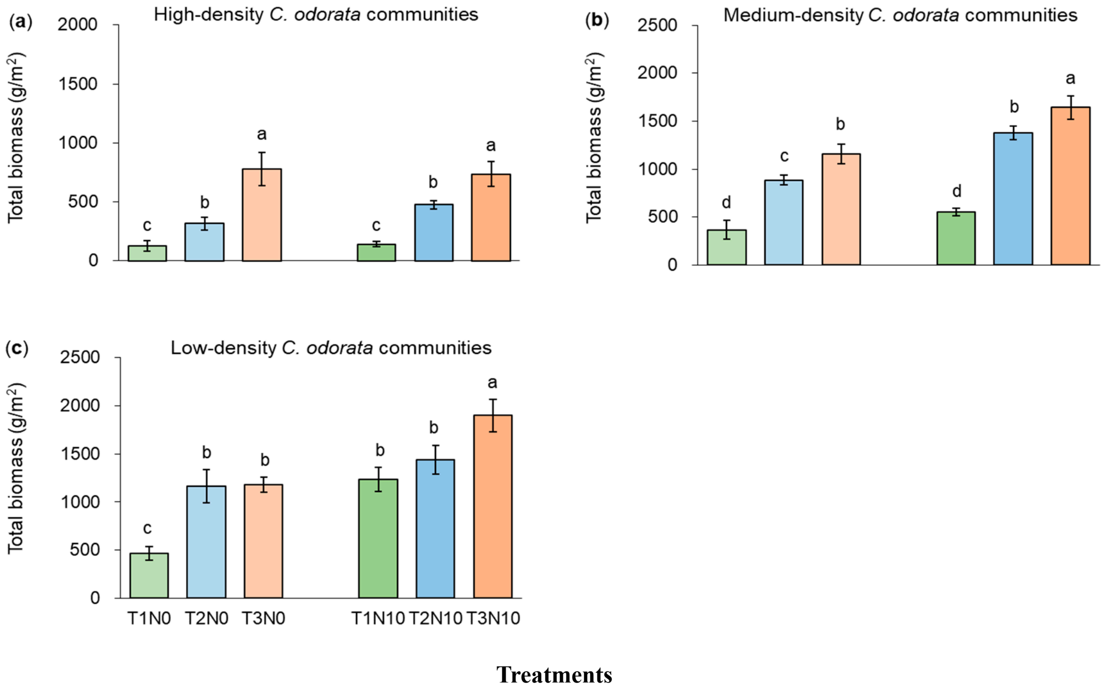

Figure S1. Total biomass of native species in high-, medium-, and low-density *C. odorata* communities (density ratios of *C. odorata* to native species were 29:7, 15:21, and 8:28, respectively) under different arrival order scenarios. T1N0: *C. odorata* arrived early without N addition; T2N0: *C. odorata* arrived simultaneously with native species without N addition; T3N0: *C. odorata* arrived late without N addition; T1N10: *C. odorata* arrived early with N addition; T2N10: *C. odorata* arrived simultaneously with native species with N addition; T3N10: *C. odorata* arrived late with N addition. Values are means ± SE of the raw data. Different lowercase letters above bars indicate significant differences among treatments ($p < 0.05$), as determined by ANOVA on square root-transformed (a), and untransformed (b, c) data, respectively.
